# HiHi fMRI: a data-reordering method for measuring the hemodynamic response of the brain with high temporal resolution and high SNR

**DOI:** 10.1093/cercor/bhac364

**Published:** 2022-09-28

**Authors:** Zoltan Nagy, Chloe Hutton, Gergely David, Natalie Hinterholzer, Ralf Deichmann, Nikolaus Weiskopf, S Johanna Vannesjo

**Affiliations:** Laboratory for Social and Neural Systems Research (SNS Lab), University Hospital Zurich, Rämistrasse 100, University of Zurich, Zurich CH-8091, Switzerland; Wellcome Centre for Human Neuroimaging, UCL Queen Square Institute of Neurology, 12 Queen Square, University College London, London WC1N 3BG, UK; Wellcome Centre for Human Neuroimaging, UCL Queen Square Institute of Neurology, 12 Queen Square, University College London, London WC1N 3BG, UK; Spinal Cord Injury Center, Balgrist University Hospital, Forchstrasse 340, University of Zurich, Zurich CH-8008, Switzerland; SCMI, Swiss Center for Musculoskeletal Imaging, Balgrist Campus AG, Lengghalde 5, Zurich CH-8008, Switzerland; Wellcome Centre for Human Neuroimaging, UCL Queen Square Institute of Neurology, 12 Queen Square, University College London, London WC1N 3BG, UK; Brain Imaging Centre, Goethe University Frankfurt, University Hospital Campus, Haus 95H, Schleusenweg 2-16, Frankfurt am Main D-60528, Germany; Wellcome Centre for Human Neuroimaging, UCL Queen Square Institute of Neurology, 12 Queen Square, University College London, London WC1N 3BG, UK; Department of Neurophysics, Max Planck Institute for Human Cognitive and Brain Sciences, Stephanstrasse 1a, Leipzig 04103, Germany; Department of Physics, Norwegian University of Science and Technology, Høgskoleringen 5, Trondheim 7491, Norway

**Keywords:** hemodynamic response, SNR, fMRI, BOLD, sampling rate

## Abstract

There is emerging evidence that sampling the blood-oxygen-level-dependent (BOLD) response with high temporal resolution opens up new avenues to study the in vivo functioning of the human brain with functional magnetic resonance imaging. Because the speed of sampling and the signal level are intrinsically connected in magnetic resonance imaging via the T1 relaxation time, optimization efforts usually must make a trade-off to increase the temporal sampling rate at the cost of the signal level. We present a method, which combines a sparse event-related stimulus paradigm with subsequent data reshuffling to achieve high temporal resolution while maintaining high signal levels (HiHi). The proof-of-principle is presented by separately measuring the single-voxel time course of the BOLD response in both the primary visual and primary motor cortices with 100-ms temporal resolution.

## Introduction

The difference between the magnetic properties of arterial and venous blood was described by Pauling and Coryell (1936). By the 1990s, advances in magnetic resonance imaging (MRI) (Lauterbur 1973) allowed to observe this endogenous contrast in vivo in humans (Ogawa et al. 1990; Turner et al. 1991; Bandettini et al. 1992; Kwong et al. 1992). Its experimental utility stems from the fact that the blood-oxygen-level-dependent (BOLD) MRI signal is a surrogate marker of the energy consumption accompanying neuronal activity (Magistretti et al. 1999).

Acquiring functional magnetic resonance imaging (fMRI) data usually utilizes echo planar imaging (EPI) (Schmitt et al. 1998), covering the human brain with 2D slices in 2–4 s (for practical ranges of in-plane resolution and slice thickness). In a recent review, Polimeni and Lewis (2021) outlined applications of sampling the BOLD response with higher temporal resolution, demonstrating how faster sampling widens the applicability of fMRI. Although multiband (MB) imaging (Larkman et al. 2001; Moeller et al. 2010) or slice number reductions (Biswal et al. 1995; Menon et al. 1998) allow for increased temporal sampling rates, these methods incur an inherent MRI signal—and hence signal-to-noise ratio (SNR)—reduction due to incomplete T1 relaxation.

**Fig. 1:**
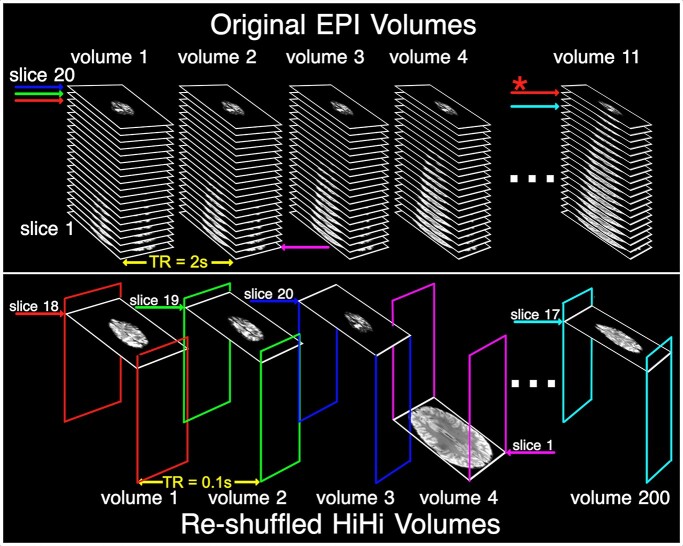
Schematic guide to the HiHi reshuffling step. On the top, 11 volumes of an EPI time series are shown. The TR is 2 s and the 20 slices in each volume are collected in ascending order. On the bottom, a pseudo time series is represented, which is initially empty but can hold 200 volumes of 20 slices each. Suppose the visual stimulus (or motor response) occurs while acquiring slice 18 of volume 1 of the original EPI data (red arrow). A copy of this slice is inserted into volume 1 (red frame) of the pseudo time series as slice 18. The subsequent EPI slice of the original time series is acquired 100 ms later (green arrow) and is inserted into volume 2 of the pseudo time series (green frame) as slice 19. Note that the 100-ms slice TR of the original EPI data becomes the volume TR of the pseudo time series. The process is continued until 200 successively acquired slices (cyan arrow) from the original time series are distributed across 200 HiHi volumes (bottom) while maintaining the slice position. After 1 visual stimulus (or motor response), there is a single slice in each volume of the pseudo time series. Next, the stimulus timing is shifted by one slice (red arrow with asterisk in volume 11), and the process is repeated for the subsequent 20 s—resulting in 2 slices per volume in the pseudo time series. After 20 stimuli (or motor responses) all slices in all volumes of the pseudo time series are populated, where the *i*th volume consists of slices that were acquired exactly (*i* − 1) * 100 ms after the visual stimulus (or motor response).

The method presented here (Fig. 1) enables sampling the BOLD response with high temporal resolution while maintaining high SNR (HiHi). As a proof of principle, we sampled the BOLD response at 100-ms temporal resolution from both the primary visual and primary motor cortices with the help of a whole-body 7T MRI scanner.

## Materials and methods

To achieve the high temporal resolution, the HiHi method employs a sparse event-related (Friston et al. 1998) stimulus paradigm during acquisition and subsequent reshuffling of the acquired EPI slices. For each stimulus repetition, the timing of the stimulus is shifted relative to the EPI acquisition to coincide with the RF excitation of a different slice. Then, by reordering the EPI slices according to their relative stimulus timing, it is possible to create a pseudo time series with high temporal resolution that covers the post stimulus time and can be considered an analog of an “epoch”—as used in electroencephalography/magnetoencephalography research. In effect, by acquiring 20 slices every 2 s, the slices in the original time series are 100 ms apart. Thus, after reordering, the volume repetition time (TR) of the HiHi time series will be identically 100 ms. The principle is illustrated in Fig. 1. Importantly, this allows the fMRI data to be acquired with a long TR (here 2 s) for a desired (potentially complete) T1 relaxation. As implemented here, 10 EPI volumes (i.e. 200 slices) were collected after each visual stimulus or motor task. Thus, in order to fill up the HiHi time series once, the original time series must contain 201 EPI volumes—i.e. 10 volumes for each of the 20 stimuli and 1 additional volume resulted from shifting the visual stimulus or motor task timing by 1 slice each time. The above block was repeated 5 times, 4 of which contained the respective task (either a visual stimulus or an instruction for a motor task), while one repeat did not—the latter of which served as a control experiment. In total, this required 1,005 EPI volumes in the original time series. We added 5 dummy volumes in the beginning and the end, resulting in a total of 1,015 EPI volumes for each of the visual and motor experiments (Table 1).

**Table 1:** Sequence acquisition parameters for the anatomical and EPI data.

Parameter	fMRI	Anatomical
TR (ms)	2,000	4,000
TE (ms)	25	2.78
Flip angle (°)	73	4 & 5
Voxel size (mm^3^)	2 × 2 × 2	0.4 × 0.4 × 0.4
FOV (phase FOV %)	180 (81%)	240 (105%)
Image volumes	1,015	1
Acquisition time (m:s)	33:58	11:02
Bandwidth/pixel (Hz)	1,860	260
Number of slices	20	480
Slice orientation	Axial (motor), coronal (visual)	Sagittal
Phase encode direction	Left/right	Anterior/posterior
Partial Fourier	Off	7/8 (slice & phase)
Sequence type	2D EPI	3D MP2RAGE
Inversion time (ms)	N/A	788 & 2,980
Coil combine mode	Sum of squares	
GRAPPA	2	

Corrections for head motion and signal drift were performed on the original data (Fig. 1, top).

The data and code are available upon request from the authors.

### Motor task and visual stimulus

The visual stimulus and the visual cues for performing the motor task were programmed in nordicAktiva (Nordic NeuroLab, Bergen, Norway) and presented on their MRI-compatible screen (40″ in diameter with 3,840 × 2,160 pixels). The participant viewed a uniform gray background (R/G/B—127/127/127) with a small white fixation cross. In the visual experiment, the stimulus was a rectangular black-and-white checker board that flickered at 8 Hz and was present for 375 ms. For the motor experiment, motor task performance was cued by giving the solid gray background a slight red tint (R/G/B—200/127/127) for 80 ms. Following this visual cue, participants squeezed a response grip (Nordic NeuroLab) once with the right index finger and thumb. In total, there were 80 visual stimuli in the visual experiment and 80 squeeze events in the motor experiment.

In the visual experiment, the presentation times of the flickering checkerboard stimulus were assumed to coincide with neuronal activity while in the motor experiment the neuronal activation was assumed to coincide with the times of the motor responses, which were slightly delayed (by ∼400–800 ms) relative to the presentation of the visual cue—as described in detail in the Data processing section below.

### Data acquisition

#### Ethical considerations

The study was approved by the Cantonal Ethics Committee of Zurich (EK-2018-00937). The experiments involved one adult male participant, who signed a written informed consent before scanning.

#### Imaging protocol

All MRI data were collected on a 7T MAGNETOM Terra scanner (Siemens Healthcare, Erlangen, Germany) and its single-channel transmit and 32ch receive head coil (Nova Medical Inc., MA, United States). The visual and motor experiments were carried out in separate sessions that took place on different days. Each experimental session comprised a 3D T1-weighted data set for anatomical guidance and fMRI data, utilizing a 2D EPI sequence (Schmitt et al. 1998). Relevant details of the acquisition protocol are listed in Table 1.

### Data processing

Prior to reshuffling, 3D rigid body motion correction and voxel-wise signal detrending with a second-order polynomial fit were performed within the original time series. In the visual experiment, involving a stimulus that the participant viewed passively, the stimulus presentation times were assumed to coincide with neuronal activity. Therefore, these times were used directly for reordering the data. In the motor experiment, identical timing was chosen for presenting the visual cues that instructed the volunteer participant to perform the motor task. The actual times when the participant performed the motor task were slightly delayed (by ∼400–800 ms) relative to the presentation of the visual cue. Each motor action could be detected specifically via the signal from the response grip that the participants squeezed. Subsequently, these response times were used for the reordering the motor task fMRI data (Fig. 1, bottom).

After reshuffling, the 4 repeats during which the respective task was performed (either visual stimulus or motor task) were averaged to improve the SNR further. The single repeat without any task was kept and used as a control experiment to ascertain that hemodynamic responses are not a false-positive side-effect of the reordering but are rather evoked by the task only.

Other variance sources (e.g. global signal fluctuations resulting from RF power instabilities) could also be corrected in the original time series. Still, signal fluctuations resulting from cardiac pulsation, breathing, or T1 relaxation effects following head motion are more challenging because these effects are usually entered in the design matrix together with the expected signal fluctuation (i.e. stimulus convolved with an assumed hemodynamic response function). However, fitting a specific hemodynamic response function to the original data also colors the time series depending on the differences between the assumed model and the actual hemodynamic response in a particular voxel (which is known to vary across voxels in a single brain and across individuals).

More generally speaking, data reshuffling alters the spatio-temporal correlations between the data points. For example, in the data acquired here, adjacent time points in the original time series will be 20 time points apart in the HiHi time series (Fig. 1). Dealing with resulting effects will require further work to test how to best address the changes in the covariance structure of the data—in particular, whether corrections should be made in the original time series, in the HiHi time series, or perhaps in both in an iterative fashion.

On the other hand, as implemented here, separate and explicit slice-time correction is not necessary in HiHi fMRI, which can be considered as an advantage because it avoids side effects from interpolation/undersampling. Rather, the reshuffling step creates EPI volumes that are “snap shots” of slices occurring at the same poststimulus (epoch) time because all slices of the *i*th volume of the pseudo time series are acquired exactly (*i* − 1)*100 ms after the stimulus or motor response. Thus, the method, as presented here, inherently eliminates the timing differences among the slices within a given volume.

## Results

**Fig. 2:**
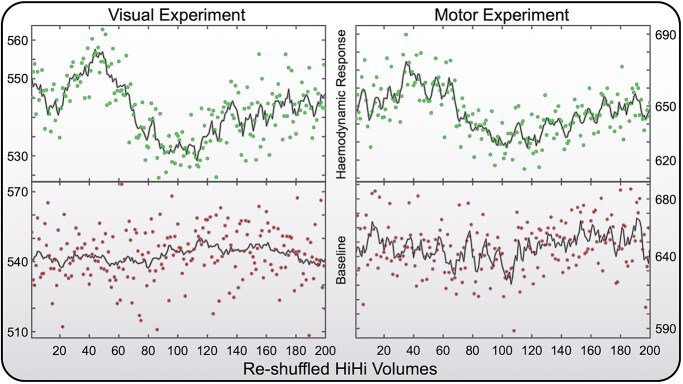
HiHi fMRI results from the primary visual and primary motor cortices. Top: BOLD response derived from two separate voxels in the primary visual cortex (left) and the primary motor cortex (right). The data displayed here are an average of four repeats of the visual stimulus or the motor task, respectively. Bottom: respective control data derived from the same voxels but only a single repetition without task performance. The single voxel temporal SNR is approximately 50 for the data on the bottom without task or averaging and approximately 100 for the task data on the top, which are a result of averaging four repeats (i.e. temporal SNR increase of sqrt(4) = 2). The contrast-to-noise of the BOLD response is approximately 3. The solid black lines serve as visual guide and represent the moving average of 5 sample points in each data set.

Figure 2 (top) displays 200 data points of the human BOLD response in the primary visual (left) and primary motor (right) cortices at 100-ms temporal resolution via HiHi fMRI. Importantly, these scatter plots provide the time course for a single voxel in each cortical area without spatial smoothing. Both BOLD responses possess unique features, such as the initial dip in only the visual response. The bottom row shows data from the same two voxels but for the runs without task performance.

## Discussion

The proposed HiHi fMRI method enables sampling of the human BOLD response with high temporal resolution (here, 100 ms) from different areas of the cortex. The method relies on reshuffling the conventionally acquired EPI slices of a sparse event-related fMRI experiment, and thus providing 2 advantages: (i) the temporal resolution of the BOLD response is not limited by the volume TR of the original time series; (ii) a long TR can be chosen, allowing for sufficient T1 relaxation, which maintains optimal SNR. The HiHi method is feasible at different field strengths and has been successfully performed separately at 3T (Nagy et al. 2008).

There are various other ways to accomplish high temporal sampling of the hemodynamic response. For example, previous investigators managed to sample it at 80–250-ms intervals by repeatedly acquiring the same single slice (Biswal et al. 1995; Menon et al. 1998). This approach presents several challenges. First, it may critically lower the SNR, as repeated RF excitations acting on the same tissue volume at such short intervals hinders the relaxation of the longitudinal magnetization, thus reducing the available signal. Assuming a brain tissue T1 relaxation time of approximately 1 s, using the optimal Ernst angle of 25° for TR of 0.1 s (as in Menon et al.) leads to a steady state magnetization that is only one-fourth of that of an experiment performed with TR = 2 s. Positioning a single slice is also challenging and the low spatial coverage reduces applicability, e.g. to some primary motor or sensory experiments. Next, when the repetition time is on the order of the T2 relaxation time of the tissue of interest (i.e. ~ 100 ms), special care must be taken that undesired echo pathways are properly spoiled. Finally, when head movement leads to new tissue entering the slice, signal increase from tissue with full T1 relaxation can manifest as a BOLD activation. To avoid these challenges, the HiHi method uses a longer TR (here: 2 s), which allows more complete T1 relaxation between RF pulses (and hence better SNR), full T2 relaxation, and can cover a larger anatomical area due to its multislice character.

Another method for improving the temporal sampling rate of the hemodynamic response is MB imaging (Larkman et al. 2001; Moeller et al. 2010; Feinberg and Setsompop 2013), which allows the acquisition of several slices almost simultaneously, thereby reducing the time needed to acquire all the slices in a single EPI volume. Although MB can provide a larger, potentially complete, coverage of the brain, the lower TR results in similar reduction on SNR as the practice of acquiring a single slice repeatedly.

HiHi fMRI has several desirable features, which complement MB imaging (Moeller et al. 2010) very well. It does not require custom MRI sequences or advanced RF pulse shapes and can thus be implemented on any scanner, e.g. at clinical sites without research agreements. Further, because HiHi fMRI does not require the simultaneous excitation and unfolding of several slices, it can be utilized for collecting a smaller number of contiguous slices (e.g. the primary visual cortex), avoiding problems such as slice-leakage artifacts or g-factor-related SNR losses in MB imaging (Xu et al. 2013). While HiHi fMRI delivers a higher temporal resolution, MB imaging provides a larger slice coverage for a given volume TR. Importantly, the two methods can be combined synergistically to simultaneously achieve whole-brain coverage, high temporal resolution, and high SNR with a significant reduction in acquisition time. In this case, the data would be collected with long TR and a modest MB factor of 2 or 3, performing the data reshuffling step (see Fig. 1) for groups of 2 or 3 slices at a time. Combined, the methods can be expected to cover a larger range of use cases in fMRI, where the specific requirements of the research question will guide whether HiHi, MB, or a combination of both would be most suitable.

Data reordering is not a new idea in MRI and with appropriate modifications, it can be applied to either *k*-space profiles (Paschal and Morris 2004) or EPI slices, as for example, in perfusion imaging (Yang et al. 2000). While the HiHi method widens the applicability of BOLD fMRI, on its own, it does not cover all potential applications. As the method requires a carefully timed stimulus or task, it is not readily applicable in resting-state studies or those with complex and extensive stimulus paradigms (Wittkuhn and Schuck 2021). Also, because the different points in the time series are compiled from distinct stimuli, the resulting BOLD response is best considered as an aggregate measure—i.e. an average response. Thus, variability in the response (e.g. due to habituation or alertness of the participant, endogenous variability of the local cortical area) will be averaged out, and the method is unsuitable for experiments where the stimulus can be shown only once. Also, care must be taken in handling the signal-dependent physiological noise component, which increases with SNR (Wald and Polimeni 2017).

Unwanted head motion can confound the results of the statistical analysis of fMRI data. Although various prospective and retrospective methods exist for realigning the image volumes (Zaitsev et al. 2015; Godenschweger et al. 2016), the resulting time series is often affected by the motion correction method used. In particular, retrospective 3D realignment of the image volumes, as performed in this work, can reduce the effective temporal resolution of the HiHi method by interpolating the signal between slices that were acquired with different post stimulus (i.e. epoch) timing. Therefore, to reach the attainable temporal resolution, it is better to properly stabilize the head during the experiment than to employ motion correction methods. For example, personalized head stabilization molds have shown promise (Power et al. 2019).

In conclusion, rapid sampling of fMRI data has exciting potential for investigating neurophysiology and behavior. Although the BOLD response is slow, aspects of its temporal evolution can be modulated by rapid stimuli and the early part of the response is thought to better reflect neuronal responses (Lewis et al. 2016; Polimeni and Lewis 2021). Other potential applications of HiHi fMRI include characterizing the BOLD response in the spinal cord and in the laminae of the neocortex. Spinal cord fMRI is severely SNR limited, and contiguous slices over small volumes are desirable, making MB imaging alone less suitable. The SNR gain provided by the HiHi method can also be traded for increased resolution, which aids the investigation of laminae in the cortex.
